# 2,3-Bis(4-ethoxy­phen­yl)quinoxaline

**DOI:** 10.1107/S1600536809052295

**Published:** 2009-12-12

**Authors:** Ping-Ping Ye, Cai-Li Zhang, Zhi-Qiang Du

**Affiliations:** a Department of Chemistry, Zhejiang University, Hangzhou 310027, People’s Republic of China

## Abstract

The title compound, C_24_H_22_N_2_O_2_, was prepared by condensation of 1,2-bis­(4-ethoxy­phen­yl)ethane-1,2-dione and 1,2-diamino­benzene. The asymmetric unit contains one half-mol­ecule, close to a twofold axis. The plane of the quinoxaline ring is twisted with respect to the planes of the two ethoxy­phenyl ring systems, exhibiting dihedral angles of 39.95 (9)°. The crystal packing is dominated by weak C—H⋯π inter­actions. No classical hydrogen bonds or stacking inter­actions are observed.

## Related literature

For applications of quinoxaline derivatives, see: Seitz *et al.* (2002 ▶); He *et al.* (2003 ▶); Dailey *et al.* (2001 ▶). For the syntheses of quinoxaline derivatives, see: Bhosale *et al.* (2005 ▶); More *et al.* (2006 ▶); Raw *et al.* (2003 ▶). For the synthesis of the title compound, see: Heravi *et al.* (2006 ▶).
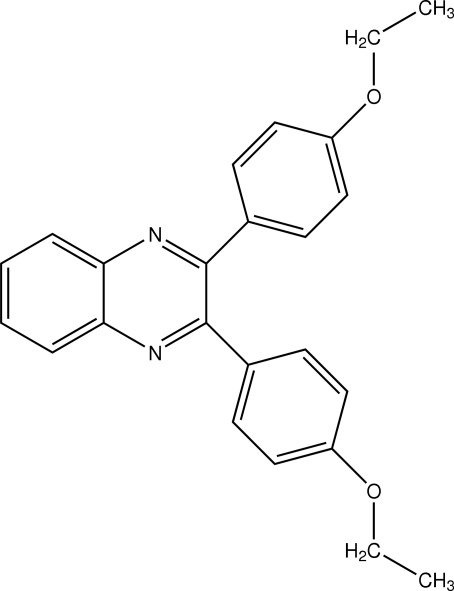


## Experimental

### 

#### Crystal data

C_24_H_22_N_2_O_2_
*M* *_r_* = 370.44
Monoclinic, 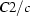

*a* = 19.4837 (18) Å
*b* = 11.2682 (11) Å
*c* = 9.2629 (9) Å
β = 100.196 (1)°
*V* = 2001.5 (3) Å^3^
*Z* = 4
Mo *K*α radiation
μ = 0.08 mm^−1^
*T* = 293 K
0.37 × 0.27 × 0.24 mm

#### Data collection

Bruker SMART CCD area-detector diffractometer
Absorption correction: multi-scan (*SADABS*; Sheldrick, 1996 ▶) *T*_min_ = 0.972, *T*_max_ = 0.981
6487 measured reflections
1743 independent reflections
1560 reflections with *I* > 2σ(*I*)
*R*_int_ = 0.028

#### Refinement

*R*[*F* ^2^ > 2σ(*F* ^2^)] = 0.035
*wR*(*F* ^2^) = 0.092
*S* = 1.03
1743 reflections
128 parameters
H-atom parameters constrained
Δρ_max_ = 0.17 e Å^−3^
Δρ_min_ = −0.12 e Å^−3^

### 

Data collection: *SMART* (Bruker, 2004 ▶); cell refinement: *SAINT* (Bruker, 2004 ▶); data reduction: *SAINT*; program(s) used to solve structure: *SHELXL97* (Sheldrick, 2008 ▶); program(s) used to refine structure: *SHELXL97* (Sheldrick, 2008 ▶); molecular graphics: *ORTEP-3* (Farrugia, 1997 ▶); software used to prepare material for publication: *WinGX* (Farrugia, 1999 ▶).

## Supplementary Material

Crystal structure: contains datablocks I, global. DOI: 10.1107/S1600536809052295/bh2259sup1.cif

Structure factors: contains datablocks I. DOI: 10.1107/S1600536809052295/bh2259Isup2.hkl

Additional supplementary materials:  crystallographic information; 3D view; checkCIF report

## Figures and Tables

**Table 1:** Hydrogen-bond geometry (Å, °)

*D*—H⋯*A*	*D*—H	H⋯*A*	*D*⋯*A*	*D*—H⋯*A*
C10—H10⋯*Cg*1^i^	0.93	2.85	3.3936 (17)	119
C11—H11*A*⋯*Cg*2^ii^	0.96	2.93	3.743 (2)	143

Symmetry codes: (i) 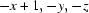; (ii) 
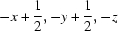
. *Cg*1 is the centroid of the N1,C1,C1′,N1′,C2 ring and *Cg*2 is the centroid of the C5–C10 ring.

**Fig. 1.**
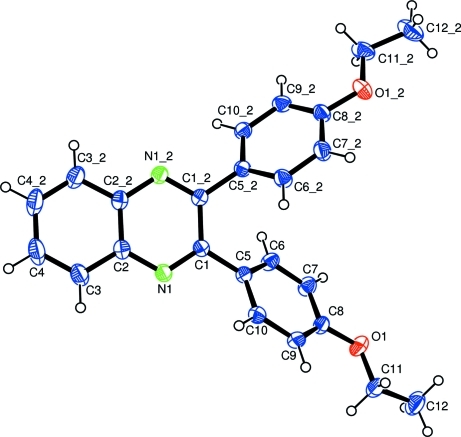
The molecular structure of the title molecule, with 40% probability displacement ellipsoids. Atoms labeled with _2 are generated by symmetry 1 - x, y, 1/2 - z.

## supplementary crystallographic information

### Comment

Quinoxaline derivatives are an important class of benzoheterocycles. They have found applications as anticancer, antiviral, and antibacterial agents (Seitz *et al.*, 2002; He *et al.*, 2003), and dyes (Dailey *et al.*, 2001). In recent years, many synthesis of quinoxaline derivatives have been reported (Raw *et al.*, 2003; Bhosale *et al.*, 2005; More *et al.*, 2006). The title compound is one of such quinoxaline derivatives. We have synthesized the title compound and report now its crystal structure.

The molecular structure of title compound is as shown in Fig. 1. The molecule lies on a twofold axis. The quinoxaline ring and two ethoxyphenyl rings are independent and planar. The quinoxaline ring is twisted with respect to the ethoxyphenyl ring with a dihedral angle of 39.95 (9)°. Packing is dominated by rather weak C—H···π interactions (Table 1). In contrast, no significant hydrogen bonds or stacking interactions are observed in the crystal structure.

### Experimental

The title compound was prepared according to the procedure reported by Heravi *et al.* (2006). A mixture of 1,2-bis(4-ethoxyphenyl)ethane-1,2-dione (1 mmol), 1,2-diaminobenzene (1 mmol), and ammonium fluoride (10% mol) in CH_3_OH (5 ml) was stirred at room temperature. The progress of the reaction was monitored by TLC. After completion, CH_2_Cl_2_ was added to the reaction mixture. The product dissolves in CH_2_Cl_2_ and the catalyst separated easily from the mixture by filtration. Solvents evaporation afforded the crude product. The solid was recrystallized from ethanol. Single crystals suitable for X-ray data collection were obtained by recrystallization from a dichloromethane-methanol mixture.

### Refinement

All H atoms were located geometrically and treated as riding, with C—H distances in the range 0.93–0.97 Å and *U*_iso_(H)=1.2*U*_eq_(parent atom) or *U*_iso_(H)=1.5*U*_eq_(parent atom) in the case of the methyl group.

### Crystal data

**Table:**

C_24_H_22_N_2_O_2_	*F*(000) = 784
*M**_r_* = 370.44	*D*_x_ = 1.229 Mg m^−^^3^
Monoclinic, *C*2/*c*	Mo *K*α radiation, λ = 0.71073 Å
Hall symbol: -C 2yc	Cell parameters from 3608 reflections
*a* = 19.4837 (18) Å	θ = 5.6–52.7°
*b* = 11.2682 (11) Å	µ = 0.08 mm^−^^1^
*c* = 9.2629 (9) Å	*T* = 293 K
β = 100.196 (1)°	Prism, yellow
*V* = 2001.5 (3) Å^3^	0.37 × 0.27 × 0.24 mm
*Z* = 4	

### Data collection

**Table:**

Bruker SMART CCD area-detector diffractometer	1743 independent reflections
Radiation source: fine-focus sealed tube	1560 reflections with *I* > 2σ(*I*)
graphite	*R*_int_ = 0.028
φ and ω scans	θ_max_ = 25.0°, θ_min_ = 2.1°
Absorption correction: multi-scan (*SADABS*; Sheldrick, 1996)	*h* = −23→22
*T*_min_ = 0.972, *T*_max_ = 0.981	*k* = −12→13
6487 measured reflections	*l* = −10→10

### Refinement

**Table:**

Refinement on *F*^2^	Secondary atom site location: difference Fourier map
Least-squares matrix: full	Hydrogen site location: inferred from neighbouring sites
*R*[*F*^2^ > 2σ(*F*^2^)] = 0.035	H-atom parameters constrained
*wR*(*F*^2^) = 0.092	*w* = 1/[σ^2^(*F*_o_^2^) + (0.0422*P*)^2^ + 0.9006*P*] where *P* = (*F*_o_^2^ + 2*F*_c_^2^)/3
*S* = 1.03	(Δ/σ)_max_ < 0.001
1743 reflections	Δρ_max_ = 0.17 e Å^−^^3^
128 parameters	Δρ_min_ = −0.12 e Å^−^^3^
0 restraints	Extinction correction: *SHELXL97* (Sheldrick, 2008), Fc^*^=kFc[1+0.001xFc^2^λ^3^/sin(2θ)]^-1/4^
Primary atom site location: structure-invariant direct methods	Extinction coefficient: 0.0289 (19)

### Fractional atomic coordinates and isotropic or equivalent isotropic displacement parameters (Å^2^)

**Table:**

	*x*	*y*	*z*	*U*_iso_*/*U*_eq_	
C1	0.46773 (6)	−0.00154 (10)	0.19704 (12)	0.0281 (3)	
N1	0.43983 (5)	−0.10164 (8)	0.14060 (11)	0.0321 (3)	
C2	0.47078 (6)	−0.20565 (10)	0.19257 (13)	0.0333 (3)	
C3	0.44262 (8)	−0.31482 (11)	0.13548 (16)	0.0459 (4)	
H3	0.4044	−0.3157	0.0594	0.055*	
C4	0.47143 (9)	−0.41910 (12)	0.19182 (17)	0.0580 (4)	
H4	0.4531	−0.4908	0.1529	0.070*	
O1	0.31520 (5)	0.41927 (8)	0.03771 (11)	0.0503 (3)	
C8	0.35080 (6)	0.31489 (10)	0.06662 (14)	0.0369 (3)	
C6	0.41928 (6)	0.19601 (11)	0.25439 (13)	0.0365 (3)	
H6	0.4395	0.1856	0.3523	0.044*	
C10	0.39626 (6)	0.12701 (10)	0.00690 (13)	0.0334 (3)	
H10	0.4003	0.0690	−0.0625	0.040*	
C5	0.42812 (6)	0.10907 (10)	0.15155 (12)	0.0294 (3)	
C7	0.38108 (7)	0.29661 (11)	0.21256 (14)	0.0402 (3)	
H7	0.3754	0.3532	0.2826	0.048*	
C9	0.35851 (6)	0.22927 (11)	−0.03675 (14)	0.0368 (3)	
H9	0.3385	0.2403	−0.1347	0.044*	
C11	0.28097 (7)	0.44243 (12)	−0.10928 (17)	0.0514 (4)	
H11A	0.2476	0.3801	−0.1428	0.062*	
H11B	0.3148	0.4455	−0.1745	0.062*	
C12	0.24435 (8)	0.55950 (14)	−0.1089 (2)	0.0689 (5)	
H12A	0.2209	0.5777	−0.2065	0.103*	
H12B	0.2778	0.6204	−0.0755	0.103*	
H12C	0.2109	0.5552	−0.0445	0.103*	

### Atomic displacement parameters (Å^2^)

**Table:**

	*U*^11^	*U*^22^	*U*^33^	*U*^12^	*U*^13^	*U*^23^
C1	0.0309 (6)	0.0289 (6)	0.0251 (6)	−0.0019 (5)	0.0067 (5)	0.0001 (4)
N1	0.0342 (5)	0.0298 (6)	0.0320 (6)	−0.0025 (4)	0.0051 (4)	−0.0004 (4)
C2	0.0394 (6)	0.0289 (6)	0.0328 (7)	−0.0020 (5)	0.0096 (5)	−0.0004 (5)
C3	0.0582 (8)	0.0346 (7)	0.0437 (8)	−0.0107 (6)	0.0053 (6)	−0.0041 (6)
C4	0.0851 (12)	0.0279 (7)	0.0607 (10)	−0.0092 (7)	0.0119 (8)	−0.0052 (6)
O1	0.0515 (6)	0.0377 (5)	0.0575 (6)	0.0140 (4)	−0.0017 (5)	0.0093 (4)
C8	0.0338 (6)	0.0303 (7)	0.0451 (8)	0.0029 (5)	0.0032 (5)	0.0078 (5)
C6	0.0426 (7)	0.0371 (7)	0.0286 (6)	0.0073 (5)	0.0030 (5)	0.0014 (5)
C10	0.0343 (6)	0.0331 (7)	0.0320 (7)	−0.0020 (5)	0.0037 (5)	−0.0010 (5)
C5	0.0285 (5)	0.0290 (6)	0.0307 (6)	−0.0010 (5)	0.0048 (4)	0.0015 (5)
C7	0.0472 (7)	0.0345 (7)	0.0383 (7)	0.0093 (6)	0.0061 (6)	−0.0027 (5)
C9	0.0365 (6)	0.0385 (7)	0.0327 (7)	−0.0014 (5)	−0.0015 (5)	0.0065 (5)
C11	0.0398 (7)	0.0482 (8)	0.0622 (10)	0.0006 (6)	−0.0024 (6)	0.0242 (7)
C12	0.0434 (8)	0.0553 (10)	0.1049 (14)	0.0103 (7)	0.0049 (8)	0.0361 (9)

### Geometric parameters (Å, °)

**Table:**

C1—N1	1.3193 (14)	C6—C7	1.3737 (17)
C1—C1^i^	1.452 (2)	C6—C5	1.3981 (16)
C1—C5	1.4871 (15)	C6—H6	0.9300
N1—C2	1.3660 (15)	C10—C9	1.3881 (17)
C2—C3	1.4111 (17)	C10—C5	1.3882 (16)
C2—C2^i^	1.414 (2)	C10—H10	0.9300
C3—C4	1.3655 (19)	C7—H7	0.9300
C3—H3	0.9300	C9—H9	0.9300
C4—C4^i^	1.406 (3)	C11—C12	1.500 (2)
C4—H4	0.9300	C11—H11A	0.9700
O1—C8	1.3676 (14)	C11—H11B	0.9700
O1—C11	1.4301 (17)	C12—H12A	0.9600
C8—C9	1.3860 (18)	C12—H12B	0.9600
C8—C7	1.3912 (18)	C12—H12C	0.9600
			
N1—C1—C1^i^	120.98 (6)	C5—C10—H10	119.2
N1—C1—C5	116.59 (10)	C10—C5—C6	117.88 (11)
C1^i^—C1—C5	122.38 (6)	C10—C5—C1	121.10 (10)
C1—N1—C2	117.95 (10)	C6—C5—C1	120.97 (10)
N1—C2—C3	119.85 (11)	C6—C7—C8	120.77 (11)
N1—C2—C2^i^	120.77 (6)	C6—C7—H7	119.6
C3—C2—C2^i^	119.33 (8)	C8—C7—H7	119.6
C4—C3—C2	120.05 (13)	C8—C9—C10	119.56 (11)
C4—C3—H3	120.0	C8—C9—H9	120.2
C2—C3—H3	120.0	C10—C9—H9	120.2
C3—C4—C4^i^	120.61 (8)	O1—C11—C12	107.47 (14)
C3—C4—H4	119.7	O1—C11—H11A	110.2
C4^i^—C4—H4	119.7	C12—C11—H11A	110.2
C8—O1—C11	118.57 (11)	O1—C11—H11B	110.2
O1—C8—C9	125.21 (11)	C12—C11—H11B	110.2
O1—C8—C7	115.51 (11)	H11A—C11—H11B	108.5
C9—C8—C7	119.28 (11)	C11—C12—H12A	109.5
C7—C6—C5	120.80 (11)	C11—C12—H12B	109.5
C7—C6—H6	119.6	H12A—C12—H12B	109.5
C5—C6—H6	119.6	C11—C12—H12C	109.5
C9—C10—C5	121.69 (11)	H12A—C12—H12C	109.5
C9—C10—H10	119.2	H12B—C12—H12C	109.5

Symmetry codes: (i) −*x*+1, *y*, −*z*+1/2.

### Hydrogen-bond geometry (Å, °)

**Table:**

Cg1 is the centroid of the N1,C1,C1',N1',C2 ring and Cg2 is the centroid of the C5–C10 ring.

**Table:**

*D*—H···*A*	*D*—H	H···*A*	*D*···*A*	*D*—H···*A*
C10—H10···Cg1^ii^	0.93	2.85	3.3936 (17)	119
C11—H11A···Cg2^iii^	0.96	2.93	3.743 (2)	143

Symmetry codes: (ii) −*x*+1, −*y*, −*z*; (iii) −*x*+1/2, −*y*+1/2, −*z*.
